# Dynamic immune response characteristics of piglets infected with *Actinobacillus pleuropneumoniae* through omic

**DOI:** 10.1186/s13568-021-01336-z

**Published:** 2021-12-24

**Authors:** Rining Zhu, Hexiang Jiang, Jun Wang, Chuntong Bao, Haiyao Liu, Fengyang Li, Liancheng Lei

**Affiliations:** 1 College of Veterinary Medicine, Jilin University, Xi’an Road, Changchun, 5333 People’s Republic of China; 2 College of Life Science and Engineering, Northwest Minzu University, Lanzhou, People’s Republic of China; 3 College of Animal Science, Yangtze University, Jingzhou, 434023 Hubei People’s Republic of China

**Keywords:** *Actinobacillus pleuropneumoniae*, Porcine pleuropneumonia, Omics, Immune response

## Abstract

Porcine infectious pleuropneumonia is characterized by a high-rate of carriage and mixed infection with other pathogens. The host immune response induced by *Actinobacillus pleuropneumoniae* (APP) is the basis for elucidating pathogenesis and controlling disease. However, there is currently no comprehensive and dynamic data characterising the host immune response. In this study, piglets were infected with APP and differentially expressed proteins of bronchoalveolar lavage fluid (BALF) and peripheral serum were identified by iTRAQ-LC-MS/MS, and differentially expressed genes of peripheral blood mononuclear cells (PBMC) by RNA-seq. The results of the integrated analysis of serum, BALF and PBMC showed significant metabolism and local immune responses in BALF, the general immune response in PBMC mainly involves cytokines, while that in serum mainly involves biosynthesis, phagosome, and complement and coagulation cascades. Furthermore, immune responses in PBMCs and serum were rapid and maintained compared to the lung where metabolism and cell adhesion activities were enriched. Some innate immunity pathways of the cellular response to ROS, neutrophil mediated immunity, granulocyte activation and leukocyte cell-cell adhesion were identified as central points, connecting multiple signaling pathways to form an integrated large network. At 24 h post-infection, 14 molecules were up regulated in BALF, 10 of which were shared with PBMC, but at 120 h, 20 down-regulated molecules were identified in BALF, 11 of them still up- regulated in PBMC. We conclude that, the immune response in the lung is different from that in blood, but there is a similarity in response in PBMC and serum.

**Supplementary Information:**

The online version contains supplementary material available at 10.1186/s13568-021-01336-z.

## Introduction

*Actinobacillus pleuropneumoniae* (APP) is the causative agent of contact-contagious porcine pleural pneumonia, which is a highly infectious respiratory disease that causes huge economic losses to the pig industry worldwide (Gómez-Laguna et al. 2014; Sassu et al. 2017). APP has high morbidity and mortality rates and a reason for the high mortality rate is an excessive cytokine-mediated inflammatory response (Auger et al. 2009; Skovgaard et al. 2009; Tisoncik et al. 2012; Reed et al. 2015). Therefore, investigation of the mechanisms of interaction between the host and the bacterium, and pathogenesis of APP contributes to preventing and controlling APP-associated diseases and the development of new vaccines and diagnostic reagents.

The host bronchoalveolar lavage fluid (BALF) is the primary response site upon APP infection, and the peripheral blood contains a large number of immune cells, which are known to be important for the clearance of pathogens (Mohammadi et al. 2014). For example, peripheral blood mononuclear cells (PBMCs) can produce a variety of cytokines including TNF-α, IL-8, IFN-γ which promotes the clearance of pathogens (Klitgaard et al. 2012; Yu et al. 2013). However, APP can induce excessive production of these inflammatory cytokines, which leads to host organ dysfunction and tissue damage (Auger et al. 2009; Skovgaard et al. 2009; Tisoncik et al. 2012; Hsu et al. 2016). At the same time, these cytokines are released into the BALF along with other inflammatory immune effector proteins, which makes BALF a good site to analyze the differential expression patterns of proteins and understand the local immunopathological process of lung tissue during APP infection (Damiele et al. 1985; Padrid 1991).

In previous studies, we investigated the transcriptome of porcine PBMCs and the proteomics of porcine serum after infection with APP (Jiang et al. 2018; Zhu et al. 2020). In order to further assess host response to APP and identify novel important target molecules, we have determined the transcriptome of PBMCs at different stages of APP infection in combination with proteomics of serum and BALF. Our approach allows identification of novel markers and provides new therapeutic targets for the prevention and treatment of APP infection.

## Materials and methods

### Bacterial strains and porcine pleural pneumonia model construction

APP serotype 5 (L20) (ATCC33377; GenBank Accession No. CP000569) was purchased from the China Institute of Veterinary Drug Control (Beijing, China) and grown in brain heart infusion broth (BHI broth) (Becton, Dickinson and Company, USA) supplemented with 15 µg/mL NAD at 37 °C for porcine challenge. Specific-pathogen-free (SPF) 35-day-old Landrace (approximately 10 kg) were infected with 3.1 × 10^9^ CFU APP by nasal dropping, and the clinical symptoms after APP infection were confirmed as reported previously (Jiang et al. 2018). The control group was challenged with PBS, after testing for the absence of APP antibodies by ELISA using APP bacterial lysates as coated antigen (Halli et al. 2014). Antibiotic-free pelleted food and water were provided ad libitum during the whole experiment. All animal experimental procedures were performed according to the Regulations for the Administration of Affairs Concerning Experimental Animals approved by the State Council of People’s Republic of China (1988.11.1).

### Sample preparation and sequencing

Samples of peripheral blood (collected from the anterior vena cava), PBMCs, BALF, and serum used in this study were collected from the APP-infected piglets as described previously (Jiang et al. 2018; Zhu et al. 2020; Baltes et al. 2001). Total RNA from PBMCs at each time point of APP infection (0 h/24 h/120 h) was harvested for RNA sequencing using the Illumina HiSeq^TM^ 4000 platform (QL Bio., Beijing, China). Samples of serum and BALF were analyzed using iTRAQ and LC-MS/MS analysis (QL Bio., Beijing, China).

### qPCR analysis

The SYBR Green method was used for qPCR detection. Primer design and synthesis were carried out by Sangon Biotech (Shanghai, China), and the primer sequence information is shown in Additional file 1: Table S1. qPCR conditions were 95 °C for 10 min, followed by 40 cycles of 95 °C for 15 s and 60 °C for another 30 s. β-actin was used as an endogenous control and the relative expression level of each gene was calculated using the 2^−∆∆Ct^ method.

### ELISA analysis

ELISA kits were purchased from Jinma Biotechnology Co., Ltd. (Shanghai, China) and the manufacturer’s instructions were followed. Each sample was tested in triplicates once on each occasion.

### Statistical analysis

Enrichment for gene ontology (GO) terms for individual comparisons was performed by the FunRich software (Benitomartin et al. 2015; Pathan et al. 2015) and Metascape analysis (Zhou et al. 2019); enrichment for KEGG terms for individual comparisons was performed by the Benjamin method using DAVID Bioinformatics Resources 6.8 (https://david.ncifcrf.gov/) (Huang et al. 2008, 2009).

## Results

### Gene ontology (GO) enrichment analysis of differentially expressed molecules (DEMs)

**Fig. 1:**
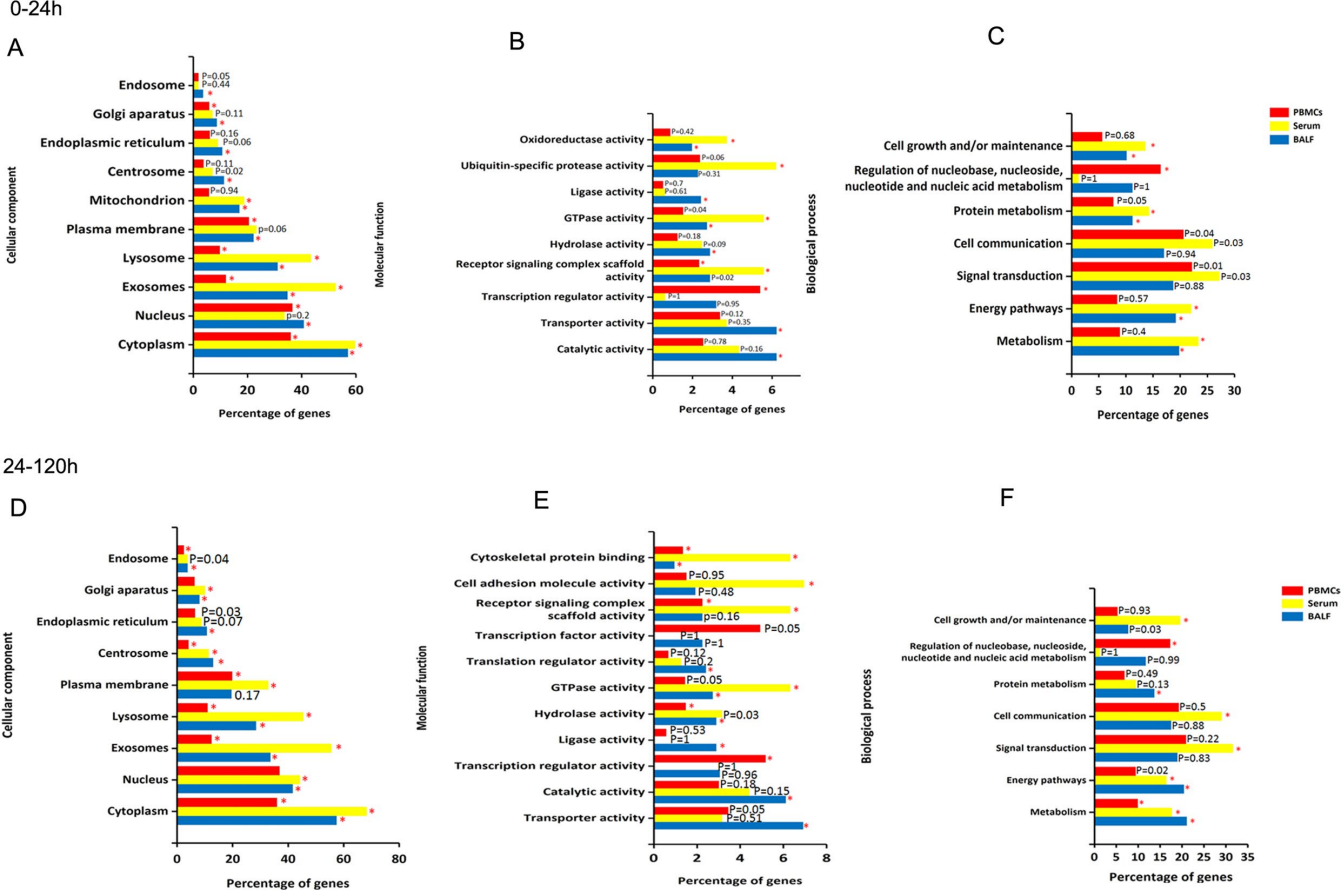
GO analysis of DEMs in PBMCs, serum, and BALF at early (0–24 h) and late (24–120 h) stage of APP infection. Comparative analysis of cell components (**A**), molecular functions (**B**), and BPs (**C**) at 0–24 h post infection; Comparative analysis of cell components (**D**), molecular functions (**E**), and BPs (**F**) at 24–120 h post infection. Red indicates PBMC; yellow indicates serum; blue indicates BALF; the X-axis indicates the percentage of DEMs enriched in this term to the total DEGs; the Y-axis indicates the GO term; significantly enriched (*P* < 0.01) GO term is denoted by *0.01 < *P*  <  0.05 or P ≥ 0.05 showed by P value

The raw transcriptome data of BALF (PXD026983) and serum (PXD017500) had been upload to PRIDE (https://www.ebi.ac.uk/pride/), and the raw transcriptome data of PMBCs had been upload to GEO (GSE179183). We first used UNIPORT (http://www.uniprot.org/uploadlists/) to convert the obtained protein identities from BALF and serum into their respective gene symbols. We defined differentially expressed genes (DEGs) and proteins (DEPs) as differentially expressed molecules (DEMs). FunRich software was used to assign the GO classification (Ashburner et al. 2000) and to analyze DEMs in PBMCs, serum, and BALF at the early infection stage (0–24 h). We found that cellular components including lysosomes, exosomes, and the cytoplasm are commonly expressed DEMs in all the three groups. Specifically, DEMs in BALF were significantly enriched in endosomes, endoplasmic reticulum and centrosomes; while in PBMC and BALF were plasma membrane and nucleus categories (Fig. 1A). There was almost no consistent DEMs in molecular function aspect in PBMCs, serum and BALF. Moreover, DEMs in PBMCs were mainly annotated as being involved in transcription regulator activity, and DEMs in serum were mainly related to ubiquitin-specific protease activity. In contrast, DEMs in BALF were involved in molecular functions such as ligase, hydrolase, catalytic, and transporter activities. Additionally, DEMs in oxidoreductase activity and TGPase activity changed consistently in both serum and BALF (Fig. 1B). In biological processes, DEMs in PBMCs were involved in regulation of nucleobase, nucleoside, nucleotide and nucleic acid metabolism; DEMs in both serum and BALF had similar changes in the cell growth and/or maintenance, protein metabolism, energy pathway and metabolism categories (Fig. 1C).

With the progression of infection (24–120 h), the significant cellular component DEMs included those in the cytoplasm, exosomes, lysosomes, Golgi and centrosomes. Specifically, DEMs in PBMCs were decreased in Golgi apparatus and nucleus, while DEMs in serum were increased in Golgi apparatus, centrosome, plasma membrane, but decreased in the mitochondrion. In BALF, there were no significant DEMs except the plasma membrane category (Fig. 1D). At 120 h post infection, the DEMs in PBMCs showed an increase in hydrolase activity, receptor signaling complex scaffold activity, and transcription regulator activity. DEMs in serum were enriched in cell adhesion molecule activity and, the changes were almost continuing from 24 to 120 h in BALF similarly. Only DEMs of cytoskeletal protein binding showed consistently up-regulation in PBMCs, serum and BALF, implying phagocytosis and cell migration for bacterial clearance is activated in both lung and periphery (Fig. 1E). Interestingly, biological process analysis showed that cell growth and maintenance, communication and signal transduction were highly active in serum, but not in PBMCs and BALF at 120 h; while material metabolism was consistently upregulated in PBMCs, serum and BALF, indicating material metabolism changes mainly during the late stage of APP infection. Moreover, regulation of nucleobase, nucleoside, nucleotide and nucleic acid metabolism was highest in PBMCs, while protein metabolism in BALF (Fig. 1F).

### Biological processes (BPs) enrichment analysis of DEMs

**Fig. 2:**
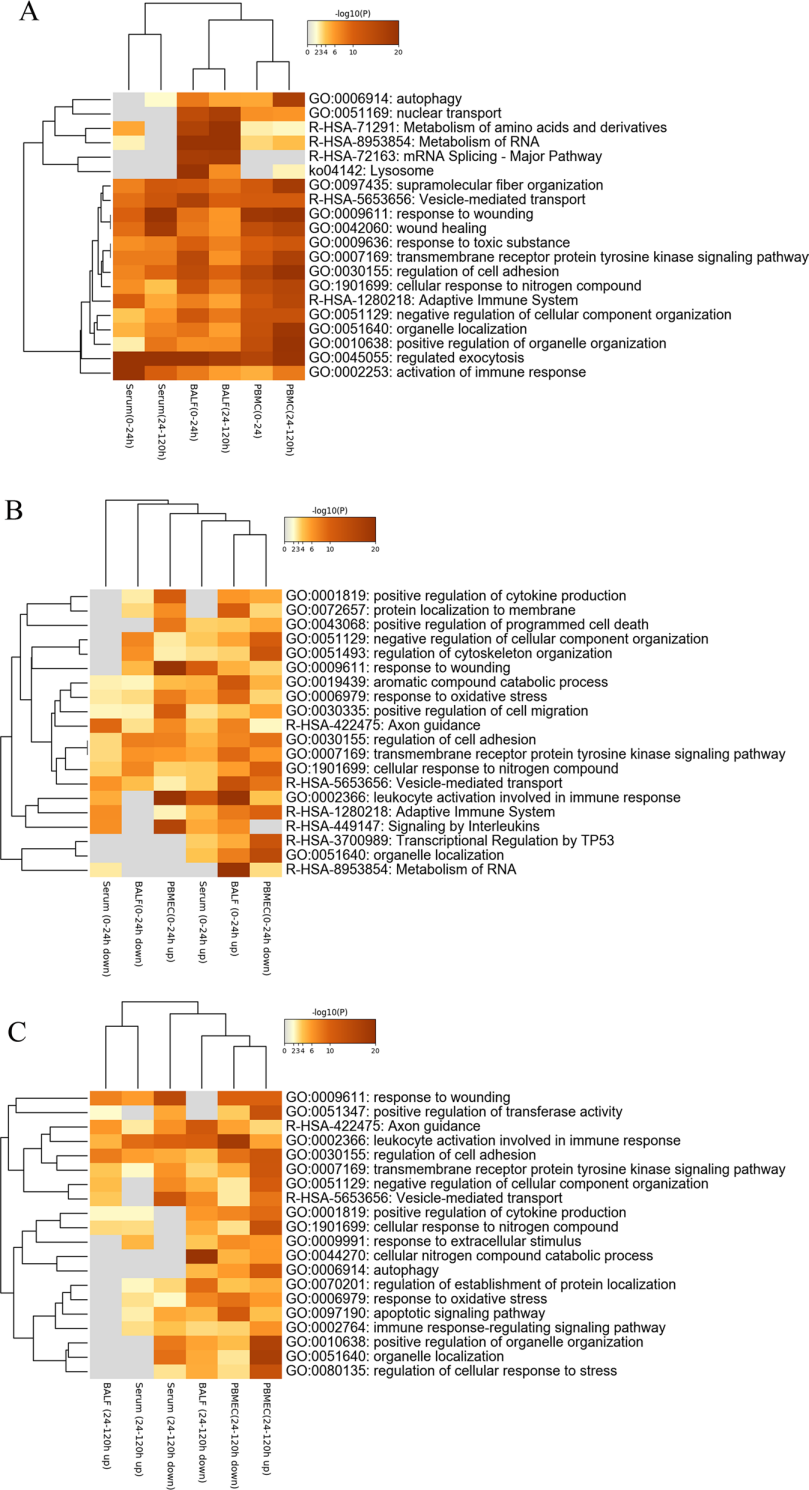
Visualizations of DEMs in PBMCs, serum, and BALF at different infection stages based on multiple gene lists. **A** Metascape visualization of DEMs during APP infection (0–24 h, 24–120 h).  Metascape visualization of up-/down-regulated DEMs at 0–24 h (**B**) and at 24–120 h (**C**). Heatmap shows the top enriched clusters, with one row per cluster, and a discrete color scale to represent statistical significance. Gray color indicates a lack of significance

Metascape analysis (Zhou et al. 2019) was used for BPs enrichment analysis of DEMs in PBMCs, serum, and BALF post infection. The results showed that the metabolism of amino acids and derivatives, and metabolism of RNA were enriched in BALF, and the lysosome is the main way for BALF to eliminate pathogens (Fig. 2A). Furthermore, we found that adaptive immunity was significantly down-regulated in the early stages of APP infection. According to our previous findings, PBMCs were one of the important sites for host immune responses, such as response to wounding, wound healing, regulation of cell adhesion, and positive regulation of organelle organization (Jiang et al. 2018). These results indicate that serum also plays an important role in the activation of immune responses (Fig. 2A). We could further see that biological pathways including metabolism, lymphocyte activation, and regulated exocytosis were present throughout the entire process of infection (0–120 h); while the immune response in serum mainly occurs in the early stage of infection (0–24 h), including activation of immune response, regulated exocytosis and adaptive immune system (Fig. 2A). Leukocyte activation involved in immune response was significantly up-regulated in the early stage of infection (0–24 h), but was gradually down-regulated by 120 h in BALF, serum and PBMCs, indicating that this pathway plays an important role in the process of host resistance to APP. PBMCs showed the strongest activities of BPs in the early stage of infection (0–24 h), not only including up-regulated signaling pathways in interleukins, response to wounding and positive regulation of cell migration, but also down-regulated signaling pathways in negative regulation of cellular component organization, regulation of cytoskeleton organization, regulation of cell adhesion, cellular response to nitrogen compound, adaptive immune system, transcriptional regulation by TP53 and organelle localization. BALF had the more obvious up-regulated BPs in terms of protein localization to membrane, aromatic compound catabolic process, response to oxidative stress, transmembrane receptor protein tyrosine kinase signaling pathway, vesicle-mediated transport, leukocyte activation involved in immune response, and metabolism of RNA, while little BPs were shown in serum (Fig. 2B). During 24–120 h post infection, more BPs of BALF were down-regulated and few were up-regulated. In contrast to BALF, more BPs were up-regulated than down-regulated in PBMCs (Fig. 2C). Together, these data demonstrate that the innate immune responses in PBMCs and serum responded rapidly and were maintained compared to the lung where metabolism and cell adhesion activities were enriched upon APP infection.

### KEGG enrichment analysis of DEMs

**Fig. 3:**
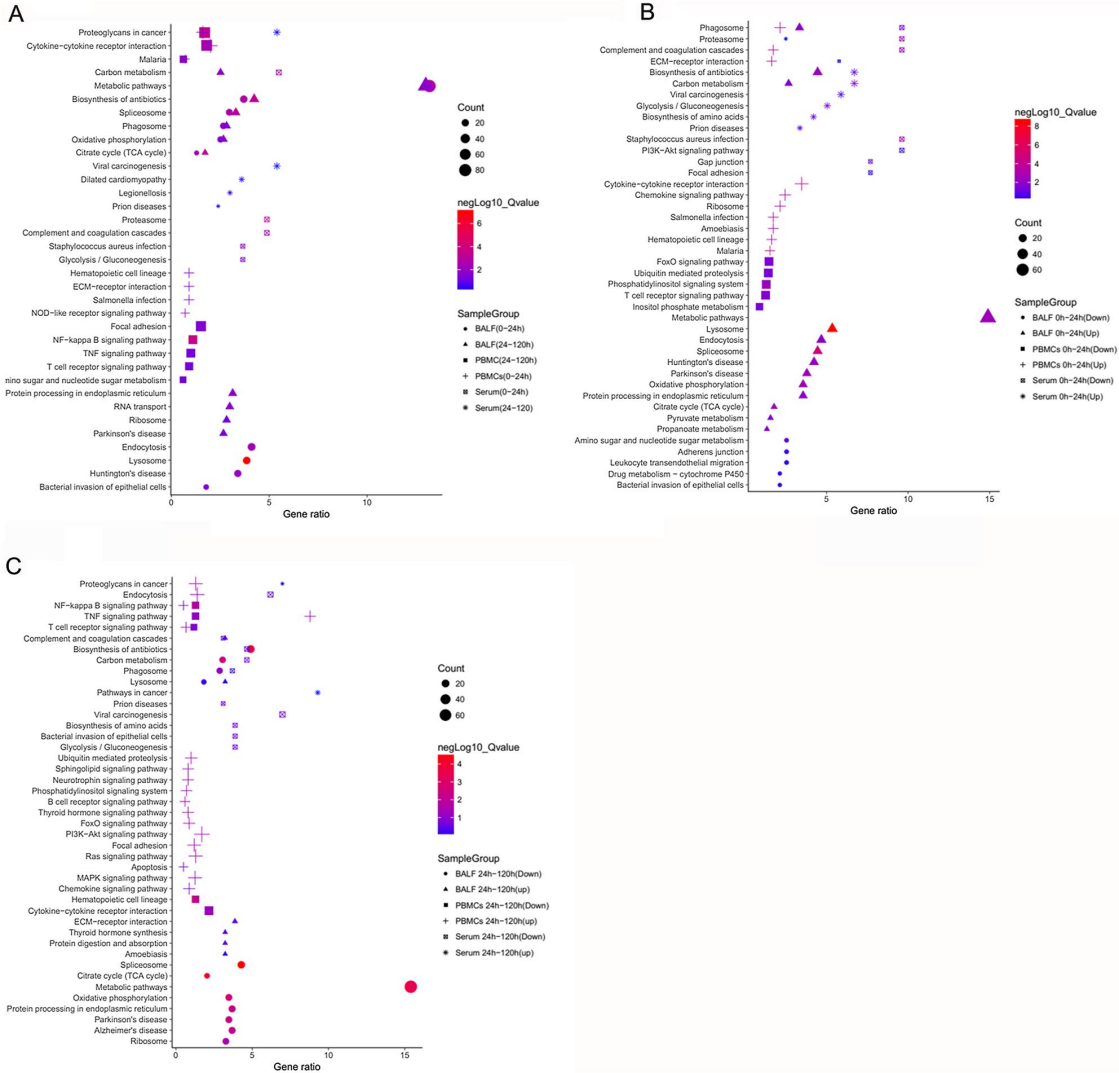
KEGG enrichment analysis of DEMs in PBMCs, serum and BALF. **A** KEGG enrichment analysis of DEMs at different infection stages (0–24 h, 24–120 h); Up-regulation and down-regulation of DEMs were analyzed by KEGG enrichment respectively at 0–24 h (**B**) and at 24–120 h (**C**). The X-axis indicates the ratio of the DEGs enriched in this pathway to the total DEGs; the Y-axis indicates the KEGG term; the counts and negLog10_qValue indicates the number and degree of enrichment of genes in a category, respectively. *negLog10_qValue > 1.3 were considered significantly enriched by the DEGs

KEGG enrichment analyses of the host immune response against APP infection were performed using Database of Annotation, Visualization, and Integrated Discovery (DAVID) Bioinformatics Resources 6.8 (https://david.ncifcrf.gov/) (Huang et al. 2008, 2009). The results from DAVID analysis are consistent with the GO enrichment analysis. During infection, BALF responds to APP mainly by induction of natural immune pathways such as phagosome, endocytosis, lysosomes and various metabolisms, including metabolic pathways, oxidative phosphorylation and TCA cycle (Fig. 3A). Moreover, these pathways were significantly up-regulated in the early infection stage (0–24 h) (Fig. 3B), while subsequently down-regulated in the late infection (24–120 h) along with metabolic pathways (Fig. 3C).

Cytokine-cytokine receptor interaction, NOD-like receptor signaling pathway, and T cell receptor signaling pathway are the main signaling pathways in PBMCs upon APP infection (Fig. 3A, B), which were in consistent with previous results (Jiang et al. 2018). Similar to that in BALF, phagosome and other pathways were also significantly enriched in PBMCs (Fig. 3B). However, the T cell receptor signaling pathway in PBMCs showed a dramatic down-regulation in the early infection stages (Fig. 3B).

The response in serum to APP infection was mainly enriched in biosynthesis and metabolism, such as carbon metabolism and glycolysis/gluconeogenesis, which was both up-regulated and down-regulated in early infection (0–24 h) and late infection (24–120 h), respectively (Fig. 3B, C). However, unlike in BALF and PBMCs, the complement and coagulation cascades and phagosome in serum showed a significant down-regulation throughout the infection (Fig. 3B). In summary, metabolic activities in lungs (based on KEGG analysis) were significantly up-regulated in the early stage, but inhibited at the later stage of infection.

### Visualization of common DEMs in serum, BALF and PBMCs during APP infection

**Fig. 4:**
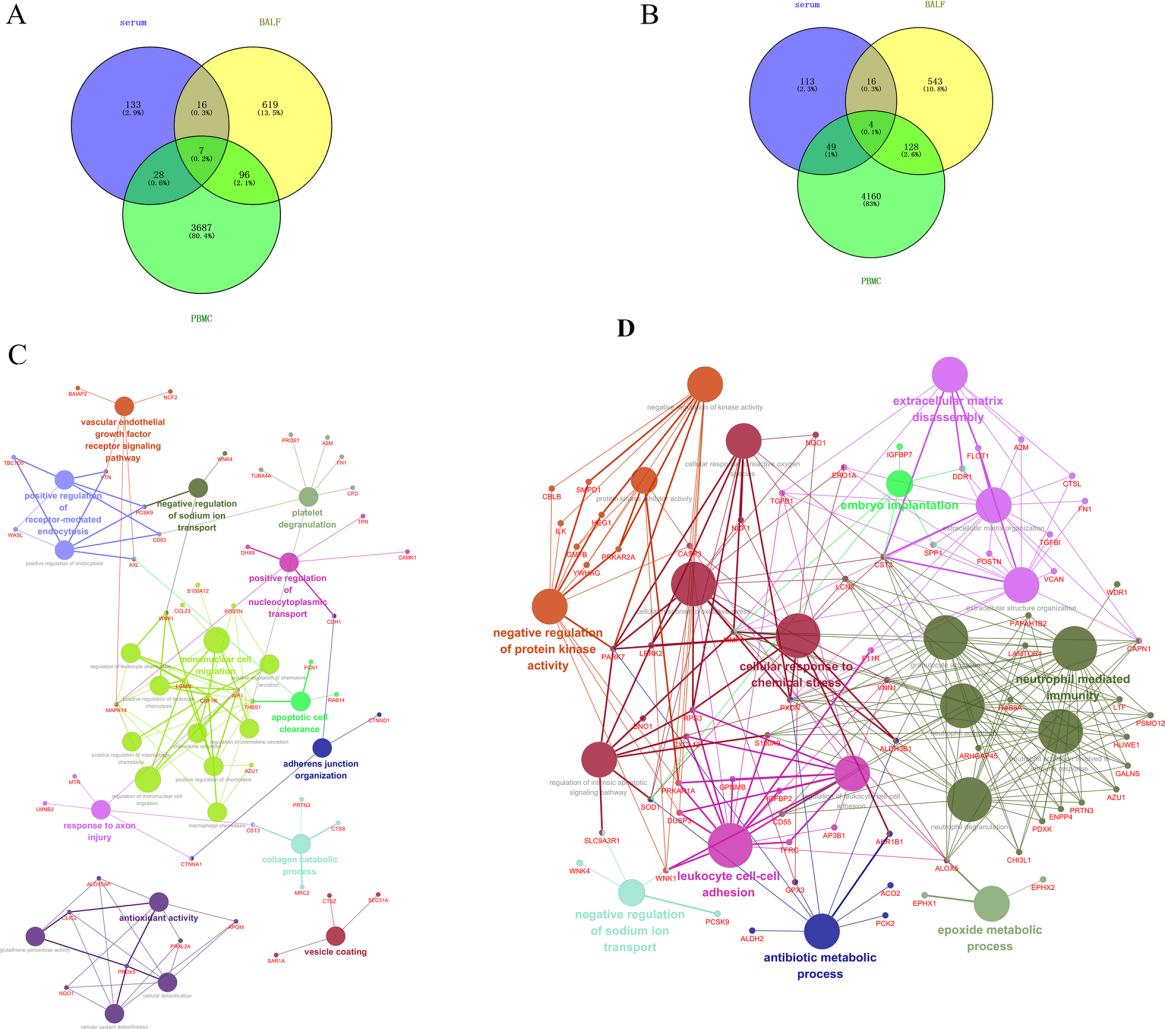
Visualization of common DEMs in serum, BALF and PBMCs during APP infection. Venn diagram shows DEMs at 0–24 h (**A**) and at 24–120 h (**B**). Cytoscape ClueGO analysis identified the link between the common DEMs and its related KEGGs at 0–24 h and at 24–120 h (**D**). Each cross-node represents a cross-talk gene (different circles indicate signal pathways where DEGs are enriched and genes on branches indicate DEMs involved in the signaling pathway)

We used Venn diagrams to identify common DEMs in serum, BALF and PBMCs. It was found that there were in total of 147 DEMs in at least two of serum, BALF and PBMC at 0–24 h, including 7 common DEMs. Twenty-three DEMs were identified in both BALF and serum at 24 h post infection, indicating that the number of DEMs in BALF (23/619 + 23 + 96) was lower than that in serum (23/133 + 28 + 23). At 120 h post infection, the number of common DEMs in both BALF and serum was similar, but the number of DEMs in BALF plus serum decreased significantly, while the number of DEMs in both PBMCs and lung were increased (Fig. 4A, B), which implied that the lung had less material exchange with serum but more with the PBMCs over the time of infection process, and immune cells in blood may participate in the immune response in lung.

We further used Cytoscape ClueGO (Bindea et al. 2009) analysis to identify cross-talk (associated with two or more pathways) DEMs, and found that these molecules were mainly enriched in mononuclear cell migration, apoptotic cell clearance and platelet degranulation (Fig. 4C). In addition, we also found that some important DEMs (i.e., THBS1, MAPK14) were involved in the regulation of different KEGGs. These data clearly showed the central points of cross-talk involves multiple BPs, e.g., the vascular endothelial growth factor receptor signaling pathway, positive regulation of receptor-mediated endocytosis, negative regulation of sodium ion transport, platelet degranulation, positive regulation of nucleocytoplasmic transport, mononuclear cell migration and antioxidant activity, several relatively concentrated interaction networks (some molecules were connected between the networks) and the main function was to initiate an immune response (Fig. 4C). However, the pathways of extracellular matrix disassembly, negative regulation of kinase activity, cellular response to reactive oxygen species, cellular response to oxidative stress, cellular response to chemical stress, neutrophil mediated immunity, granulocyte activation and leukocyte cell-cell adhesion had become the central points at 120 h post infection, and many molecules, such as CST3, A2M, RAB6A, LCN2, TGFB1, CASP3, CXCL12 were connecting points among these important pathways, connecting multiple signaling pathways to each other and forming a complex and integrated network. Moreover, innate immunity of PMNs (polymorphonuclear neutrophils) related with the anti-infection activity, complement activation, immune cell migration, immune cell stress response, antibiotic metabolism was highly active (Fig. 4D).

### Metascape analysis of the interactions between common DEMs involved in APP infection

**Fig. 5:**
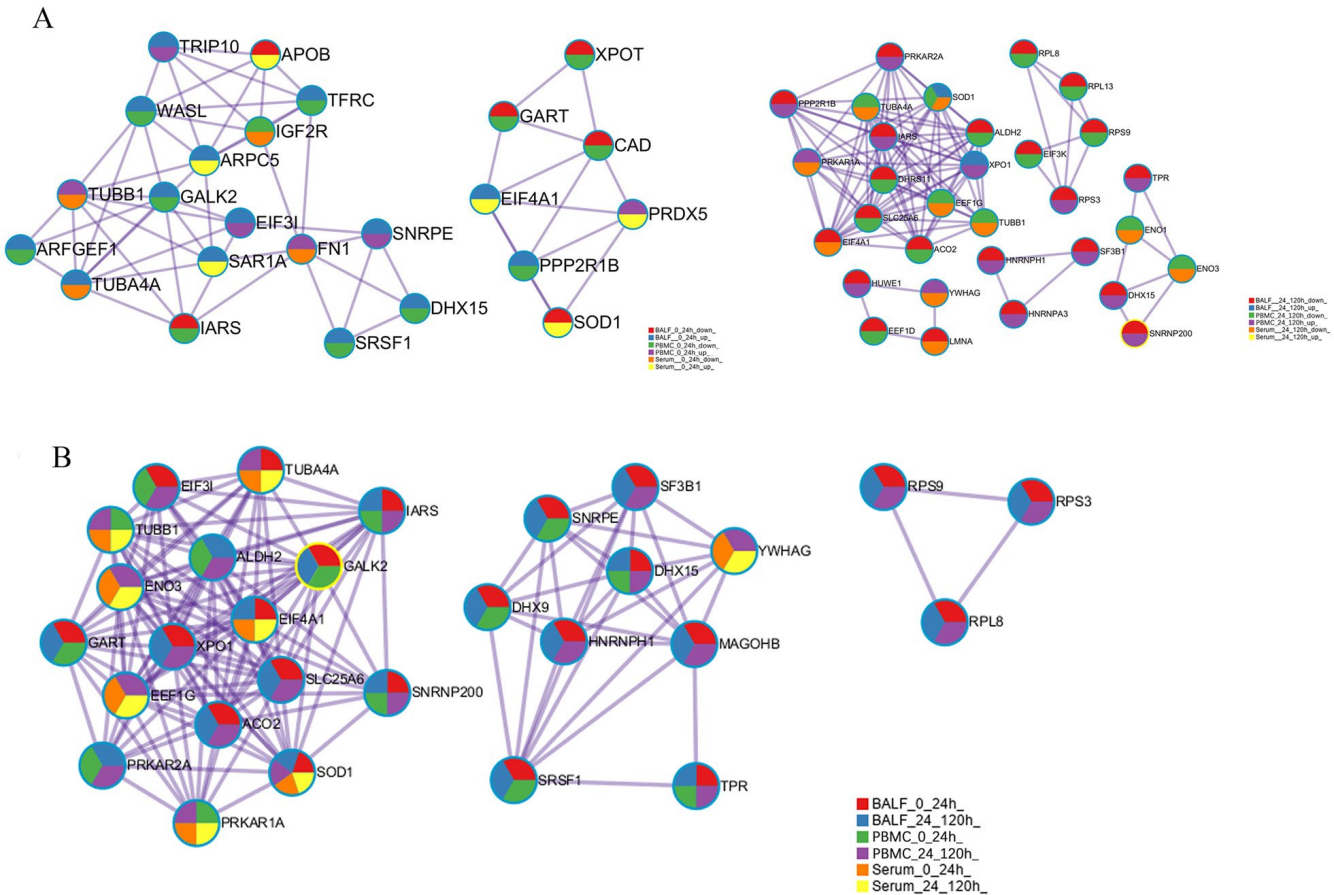
Metascape analysis of the interactions between common DEMs involved in APP infection. **A** Network nodes from up-/down-regulation of DEMs were displayed as pies. **B** Network nodes from different infection stages (0–24 h, 24–120 h) are displayed as pies. Color code for pie sector represents a gene group from which the gene is derived and is consistent with the colors used for legend

The main interaction molecules in BALF, PBMCs and serum were analyzed systematically and a large number of interactional molecules were found. At 24 h post infection, 14 molecules (TRIP10, WASL, TFRC, ARPC5, GALK2, EIF3I, SNRPE, FN1, ARFGEF1, TUBA4A, DHX15, SRSF1, EIF4A1 and PPP2R1B) were up-regulated in BALF, 10 of which were also dramatically changed in PBMCs, but only 3 of them were up-regulated. Moreover, only six (APOB, IARS, XPOT, GART, CAD, SOD1) were down-regulated in BALF, and 4 (IARS, XPOT, GART, CAD) in PBMCs. At 120 h, the up-regulated molecules in BALF were significantly reduced, only two (SOD1 and XPO1) found; while 20 down-regulated molecules (including PRKAR2A, PPP2R1B, IARS, ALDH2, DHRS11, and SLC25A6) were found in BALF, 11 of which were also up-regulated in PBMCs, and nine showed the same trend as in BALF (Fig. 5A). These data indicate that the immune response in lung is different from that in blood.

The common interaction molecular analysis of BALF and serum showed that there were four up-regulated molecules (ARPC5, SAR1A, TUBA4A, EIF4A1) found in BALF at 24 h, three of which (ARPC5, SAR1A, EIF4A1) were also up-regulated in serum; but two down-regulated molecules (APOB and SOD1) in BALF were up-regulated in serum. At 120 h, there were two up-regulated molecules (SOD1 and XPO1) in BALF. XPO1 was also up-regulated in PBMCs, while SOD1 was down-regulated. There were two down-regulated molecules (EIF4A1, LMNA) both in BALF and serum, and no up-regulated molecules in serum at 120 h (Fig. 5A). Thus, there were in total of eight common interaction molecules (ARPC5, SAR1A, TUBA4A, EIF4A1, APOB, SOD1, EIF4A1, LMNA) found in both BALF and serum, with only three (ARPC5, SAR1A, EIF4A1) having the same uptrend. Collectively, the data showed that there were significant differences in immune response between lung tissue and serum.

There were few DEMs in PBMCs. Three DEMs (TUBB1, FN1, PRDX5) were up-regulated in PBMCs at 24 h, of which PRDX5 was up-regulated, while TUBB1 and FN1 were down-regulated in serum. Only IGF2R was down-regulated both in PBMCs and serum. At 120 h, both PRKAR1A and YWHAG were up-regulated in PBMCs and down-regulated in serum. Six molecules (TUBA4A, SOD1, EEF1G, TUBB1, ENO1, ENO3) were down-regulated in both PBMCs and serum (Fig. 5A). Overall, there are only 12 coexisting molecules, with eight of them have a similar change trend.

The proportion of the main DEMs in BALF, PBMC and serum was analyzed. It was found that there were less interactive DEMs in the early stage of infection (24 h), with 22 in BALF (red), 14 in PBMC (green), and eight in serum (orange), among which 10 molecules (red + green) were common in both BALF and PBMC. In the late stage of infection (120 h), complex networks were formed, and the number of related molecules increased. There were 22 (blue) in BALF, 21 in PBMCs (purple), and eight in serum (yellow), of which 16 (blue + purple) were common in BALF and PBMCs. Notably, SOD1 is an important interaction molecule that exists both in BALF, PBMC and serum, and participates in early and late stages of infection, followed by PRKAR1A, IARS, SNRNP200, and DHX15 (Fig. 5B). These molecules interact with other DEMs during APP infection, which may play an important role in the host’s immune response.

### Key pathway node molecule detection and confirmation in the network of serum, BALF and PBMCs during APP infection

**Fig. 6:**
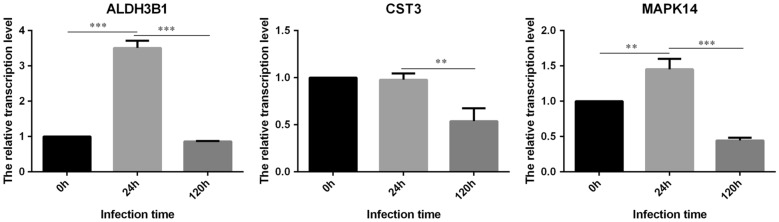
PBMC analysis of genes of interest by qPCR. Levels of Aldh3b1, Cst3 and Mapk14 mRNAs were detected in PBMCs by qPCR. Aldh3b1 mRNA level was significantly increased only at 24 h; while Cst3 mRNA significant decrease at 120 h post infection compared to the control, which were consistent with the results of PBMC transcriptome analysis. Mapk4 mRNA level was significantly increased at 24 h and decreased at 120 h, being consistent with the results of the omics data. “*” represents p < 0.05, “**” p < 0.01, “***” p < 0.001

According to the data integration analysis of serum, BALF and PBMCs, many signaling pathways involved in innate immunity were closely correlated. In order to verify the results of omics network regulation, we selected DEMs including MAPK14, ALDH3B1 and CST3 (Fig. 4C, D) to detect their mRNA levels in PBMCs by qPCR, and TUBA4A, SOD1 and EIF4A (Fig. 5A, B) to detect their protein contents in serum and BALF by ELISA. The results showed that the mRNA level of MAPK14, namely mitogen-activated protein kinase 14, was significantly increased at 24 h and decreased at 120 h after APP infection compared to the control group. This is consistent with the results of the omics data. From the network analysis of the omics, it can be seen that MAPK14 connects eight pathways at 24 h, including vascular endothelial growth factor receptor (VEGFR) signaling pathway, positive regulation of nucleocytoplasmic transport, regulation of leukocyte chemotaxis, positive regulation of leukocyte chemotaxis, mononuclear cell migration, regulation of mononuclear cell migration, and macrophage chemotaxis. Positive regulation of chemotaxis is an important pathway. However, the difference was not significant at 120 h and was not screened out in the pathway association analysis, which is consistent with the gradual remission of systematic inflammation in the late stage of APP infection (Fig. 6).

The mRNA level of ALDH3B1 (aldehyde dehydrogenase family 3 member B1) was significantly increased in PBMCs at 24 h, but not at 120 h compared to control, which was consistent with the results of PBMC transcriptome analysis. CST3, also known as Cystatin-C, is an inhibitor of cysteine protease that belongs to cystatin superfamily. qPCR found no significant difference in gene expression at 24 h, but there was a significant decrease at 120 h post infection compared to the control, which was consistent with the results of transcriptome analysis (Fig. 6).

**Fig. 7:**
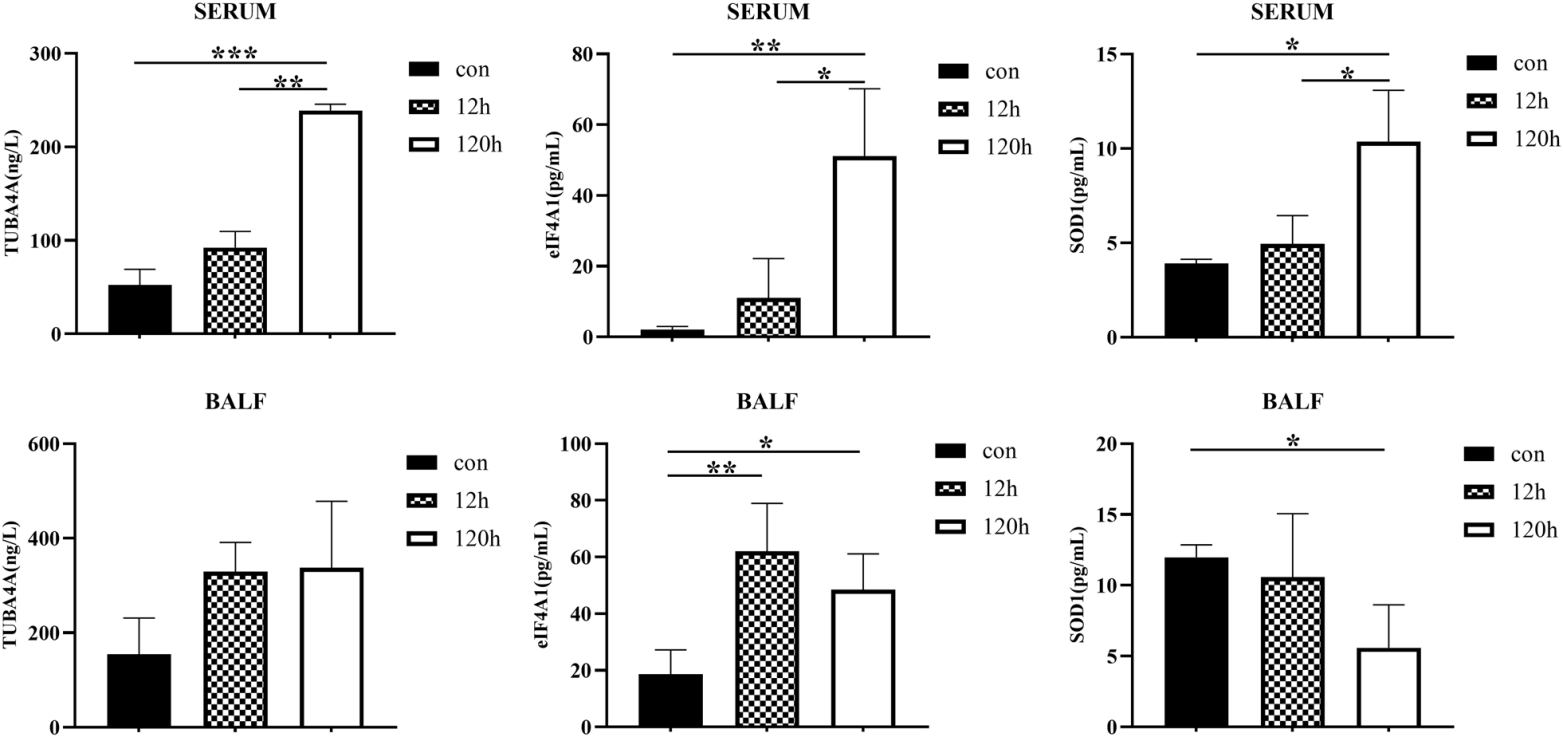
ELISA analysis of proteins of interest in BALF and serum. TUBA4A, SOD1 and EIF4A proteins in serum and BALF samples were analyzed by ELISA at 12 h and 120 h post infection. The level of TUBA4A, SOD1 and EIF4A in BALF were consistent with the results of omics analysis (12h/0h and 120h/12h). The level trends of TUBA4A, SOD1 and EIF4A in serum were also consistent with the omics data only at 12 h, no difference or increase trend at 120h/12 h in serum. “*” represents p < 0.05, “**” p < 0.01, “***” p < 0.001

For the ELISA detection of TUBA4A, SOD1 and EIF4A in serum and BALF, we only analyzed samples from 12 to 120 h post infection due to the shortage of the same batch of samples for omics detection. We found that the level of TUBA4A, SOD1 and EIF4A in BALF were consistent with the results of omics analysis (12 h/0 and 120 h/12 h) (Fig. 5A, B). The level trends of TUBA4A, SOD1 and EIF4A in serum were also consistent with the omics data only at 12 h, no difference or increase trend at 120 h/12 h in serum by ELISA assay. It was not similar with that in serum at 120 h/24 h, which might be due to the different sampling time points for ELISA (12 h) and for the omics analysis (24 h), that is to say, these proteins levels may still be rising at 12 h, higher at 24 h, so 120/24 h decreased than 120 h/12 h (Fig. 7).

## Discussion

In this study, we carried out omics analyses of BALF, PBMCs, and serum from APP infected-piglets. It was found that BALF is the main site of metabolism during the whole infection process, and the clearance of APP in lung is mainly associated with natural immune processes such as phagosome, endocytosis and lysosomes. The general immune response mainly involves the production of cytokines in PBMC, and biosynthesis, phagosome, and complement and coagulation cascades in serum. Furthermore, immune responses in PBMCs and serum were rapid and maintained compared to the lung, and innate immunity pathways of cellular response to ROS, neutrophil mediated immunity, granulocyte activation and leukocyte cell-cell adhesion as the central points to connect multiple signaling pathways to form an integrated large network. The immune response in lung is obviously different from that in blood, but their are similar trends in PBMCs and serum.

Our findings provide a comprehensive understanding on the response of lung and blood in APP infected piglets, which is extremely useful for clinical treatment. Based on the previous studies by Yu  et al.  (2013), who also identified a number of biological processes related to metabolism using transcriptional profiling of hilar nodes from pigs infected with APP, including the citrate cycle, glycolysis/gluconeogenesis, and fatty acid metabolism, and by Kim et al. (2019) who found the signaling pathways were enriched in the categories apoptosis, nuclear receptor and cellular immune response when the response of an immortalized porcine alveolar macrophage cell line was examined to App exotoxins by transcriptional analyses, we not only mutually confirmed these results, but also extended them to other body locations.

Moreover, previous studies in our laboratory have demonstrated that DEGs of PBMCs after APP infection were significantly enriched in some autoimmune disease pathways, such as rheumatoid arthritis (Jiang et al. 2018). The activated T cells of rheumatoid arthritis are dependent on glycolysis, which produces less ATP and more biomolecules that support cell division, but they lack the key signaling molecules, e.g., ROS, that control their own behavior, resulting in progression of inflammation (Perl 2017). Inflammation is the basis of many diseases, and now more and more evidence suggest that regulation of cell metabolism can alleviate inflammation (Abboud et al. 2018; Choi et al. 2018). For example, we showed that aconitase 2 (ACO2) was significantly reduced in the late infection stages (24–120 h) in PBMCs and BALF. ACO2 is an enzyme involved in the second step of the TCA cycle. Tsui et al. (2013) has shown that hypoxia can up-regulates the gene expression of mitochondrial ACO2. However, ACO2 reduced oxidant-induced alveolar epithelial cell (AEC) apoptosis (Kim et al. 2014). Therefore, ACO2 may be a regulatory molecule of the host under oxidative stress. The research by Mangialasche et al. (2015) also confirmed that ACO2 activity was reduced in peripheral lymphocytes of subjects with Alzheimer’s disease and mild cognitive impairment which correlates with antioxidant protection, and proposed ACO2 as an important marker of AD progression. Further studies are warranted to verify the role of ACO2 in the process of APP infection during lung injury. Therefore, studying the role of BALF metabolism during APP infection may lead to developments in the control and prevention of pleuropneumonia in piglets.

Our omics data indicates that the biosynthesis, complement and coagulation cascades pathways are significantly enriched in serum, which may be the primary pathway for the serum to eliminate APP. However, we found that proteins associated with these pathways were significantly reduced during infection. Resistance to host serum is an important mechanism for pathogens to evade host immunity. Relevant studies have reported that bacterial pathogens can evade host immune responses using the complement system (Qin et al. 2015). For example, binding to immunoglobulin G (IgG) and immunoglobulin M (IgM) may prevent the activation of the classical complement pathway by blocking the Fc binding site for Clq (Abdullah et al. 2005; Leo et al. 2011). In addition, the formation of membrane attack complexes (MACs) is prevented by inhibiting C5b-C7 complex formation and C9 polymerization (Singh et al. 2014; Attia et al. 2006). The resistance of APP to complement cytotoxicity can mainly be attributed to the capsular polysaccharide (CPS) and/or LPS (Bossé et al. 2002). In addition, genes associated with urease synthesis of APP might be related to the inactivation of the complement cascade for immune evasion (Bossé et al. 1997). However, there is no report on how APP uses the complement system to evade the host immune response, which needs to be further studied. Our data suggest that Fc γ R-mediated phagocytosis may be involved in the regulation of immune responses during APP infection. In our study, the protein expression levels of actin-related protein 2/3 complex subunit 5 (ARPC5) and neural Wiskott-Aldrich syndrome protein (WASL), both of which are involved in Fc γ R-mediated phagocytosis, and bacterial invasion of epithelial cells and *Salmonella* infection (Kolb-Mäurer et al. 2001), were significantly increased in BALF and serum in the early infection stage. Humphries et al. also found that vaccinia virus enhances its cell-to-cell spread by inducing Arp2/3-dependent actin polymerization, and WASL participates in Fc γ R-mediated phagocytosis during infection (Humphries et al. 2014). Moreover, we found that TUBA4A and TUBB1, both of which can interact directly with ARPC5 and WASL and participate in phagosome biological processes, were significantly down-regulated in serum. Together, these results suggest that APP may inhibit the ability of serum to clear pathogens through biological pathways such as complement and coagulation cascades and Fc γ R-mediated phagocytosis.

Through omics analysis, PBMCs were identified as a major source for the production of cytokines during the whole infection process. The cytokine-cytokine receptor interaction and chemokine signaling pathways of PBMCs were significantly up-regulated in the early stage infection (0–24 h). Excessive production of inflammatory cytokines is one of the causes of lung injury (Auger et al. 2009; Reed et al. 2015). In previous studies, the involvement of NF-κB signaling pathway in the regulation of APP exotoxin ApxI-induced IL-1β, IL-8, and TNF-α production in porcine alveolar macrophages (PAM) was demonstrated (Hsu et al. 2016). Moreover, we have also found other molecules that play an important role in APP pathogenesis, such as TRIP10, IGF2R, EIF3I. High levels of EIF3I (also called TRIP-1) reduced collagen contractility in adult fibroblasts, while the down-regulation of EIF3I expression in primary human lung fibroblasts can enhance resistance to apoptosis and collagen contraction ability (Nyp et al. 2014; Navarro et al. 2009). Therefore, EIF3I may be an important molecule in APP-induced lung injury.

The characteristics and functions of mammalian ALDH3B1-like proteins have not been reported, but it has been speculated that human and rat ALDH3A-like proteins are involved in the detoxification of endogenous and exogenous acetaldehyde (Holmes 2010). It was reported that the high expression of ALDH1 is related to the chronic infection of buffalo mammary gland, which can indicate the degree of disease progression (Thakur et al. 2019). High expression of ALDH1 is also a characteristic indicator of malignant transformation degree of ovarian cancer (Ruscito et al. 2018). The whole network analysis of our data showed that ALDH3B1 did not appear in any pathway networks at 24 h, but appears at 120 h, becoming the core molecule connecting seven pathways including cellular response to chemical stress, granulocyte activation, neutrophil activation, neutrophil degranulation and activation involved in immune response, neutrophil mediated immunity, antibiotic metabolic process, and cellular response to oxidative stress. These results suggest that ALDH3B1 may be a key innate immune response molecule involved in neutrophil activation, regulation of antimicrobial metabolism and antioxidant stress during APP infection.

CST3 can be continuously transcribed and expressed in all nucleated cells. However, the level of CST3 in serum in patients with emphysema caused by smoking and in elderly men with respiratory disorders and lung diseases was significantly increased (Rokadia et al. 2012; Zhang et al. 2014). In this study, omics data analysis showed that CST3 connected two pathways within the 24 h response to axon injury and collagen catabolic process, twelve pathways at 120 h, including extracellular matrix disassembly, extracellular matrix organization, extracellular structure organization, neutrophil mediated immunity, neutrophil activation involved in immune response, granulocyte activation, neutrophil activation, neutrophil degranulation, cellular response to reactive oxygen species, cellular response to chemical stress, cellular response to oxidative stress, and embryo implantation. These pathways are mainly involved in innate immune responses, indicating that the immune cells such as PBMCs quickly responded in the early stage of infection, resulting in a large transcription of CST3, which is involved in regulating many pathways of innate immunity and infection control. Elevated CST3 levels may be a marker of impaired lung function. In the late stage of infection, the progression of the disease was impaired by effective elimination of bacteria via innate immunity processes, and the transcription of CST3 was decreased at this time point.

This omics study systematically summarized the possible interrelationships between the immune response signaling pathways and identified important molecules that are involved in the whole process of host anti-APP infection. It is shown that the immune response in lung is quite different from that in blood, there was a similar trend in PBMCs and serum. Metabolic activities in lungs, based on KEGG analysis, were found significantly up-regulated in the early stage but inhibited at the later stage of infection. At the same time, it is suggested that APP may evade or suppress the host immune response through one or more pathways during infection, such as complement and coagulation cascades, and also apoptosis. Few studies have reported simultaneous measurement of host responses in different compartments, and ours provides novel insights into differential gene and protein expression, and identifies new directions for future research.

## Supplementary Information

**Additional file 1: Table S1.** The qPCR primer information used in this study.

## Data Availability

The data on which the conclusions are made are all presented in this paper. The raw transcriptome data of BALF (PXD026983) and serum (PXD017500) had been upload to PRIDE (https://www.ebi.ac.uk/pride/), and the raw transcriptome data of PMBCs had been upload to GEO (GSE179183).

## Abbreviations

APP: *Actinobacillus pleuropneumoniae*
PBMCs: Peripheral blood mononuclear cells
BALF: Bronchoalveolar lavage fluid
DEG: Differentially expressed genes
DEP: Differentially expressed proteins
DEM: Differentially expressed molecules
GO: Gene ontology
KEGG: Kyoto Encyclopedia of Genes and Genomes
DAVID: Database of annotation, visualization, and integrated discovery
