# Supplementary material for: Dynamic immune response characteristics of piglets infected with Actinobacillus pleuropneumoniae through omic
Source: AMB Express. 2021 Dec 24;11:175. doi: 10.1186/s13568-021-01336-z (PMC8709809; doi:10.1186/s13568-021-01336-z)
Supplement: Supplementary file 1 — Additional file 1: Table S1. The qPCR primer information used in this study. [file 13568_2021_1336_MOESM1_ESM.docx]

| Gene name | Primer name | Primer sequence |
| --- | --- | --- |
| MAPK14 | MAPK14-F | TCTACCAGATTCTCCGAGGTCTCAAG |
|  | MAPK14-R | TCTTCAGCTCACAGTCTTCATTCACAG |
| ALDH3B1 | ALDH3B1-F | GCCCATCCTGCCCATTCTGAAC |
|  | ALDH3B1-R | CCACCTGGCTGTTGTTGGAGAAG |
| CST3 | CST3-F | GTGGCGGGGATGAACTACTTCTTG |
|  | CST3-R | GGGACGGTGTAAACTTGGAAGGAG |
| β-actin | β-actin-F | TCCACGAAACTACCTTCAACTC |
|  | β-actin-R | GATCTCCTTCTGCATCCTGTC |

Table S1. The qPCR primer information used in this study
